# Supplementary material for: Polyphenols-Rich Fraction from Annona muricata Linn. Leaves Attenuates Oxidative and Inflammatory Responses in Neutrophils, Macrophages, and Experimental Lung Injury
Source: Pharmaceutics. 2022 May 31;14(6):1182. doi: 10.3390/pharmaceutics14061182 (PMC9228609; doi:10.3390/pharmaceutics14061182)
Supplement: Supplementary file 1 [file pharmaceutics-14-01182-s001.zip › pharmaceutics-1642787-supplementary.pdf]

High-Performance Liquid Chromatography - Electrospray Ionization-tandem Mass Spectrometry (HPLC-ESI/MS^n)

The HPLC-ESI/MS^n analysis was done on the Agilent Infinity 1260 coupled with a High-Resolution Mass Spectrometry Agilent 6520 B equipped with Quadrupole Time of Flight (QTOF) and electrospray ionization (ESI), according to the methodology described by Justino *et al*. 2018. The extract and fractions were injected on an Agilent Zorbax C18 column (100 mm x 2.1 mm, 2.7 µm) at 1.0 mg/mL. The mobile phase consisted of water acidified with formic acid (0.1 % v/v) (A) and methanol (B), as follows: 2% B (0 min), 98% B (0-15 min); 98% B (15-17 min); 2% B (17-22 min) at a flow 0.4 mL/min. High purity nitrogen (N_2) was used as drying gas (8.0 L/min) and as nebulizing gas at pressure of 58 PSI. The nebulizer temperature was 220 ºC with a potential of 4.5 kV used on the capillary. The mass spectra of the ions (MS and MS^2) were acquired in negative mode in the range of *m/z* 100–1000. Different collision energies were adjusted automatically according to the precursor *m/z* to obtain the fragmentation spectra (MS^2). Identification of compounds in each sample was performed by high resolution mass and fragmentation spectra MS^2 analysis, comparing with data from the literature and METLIN metabolite database.

HPLC-ESI/MS^n analysis

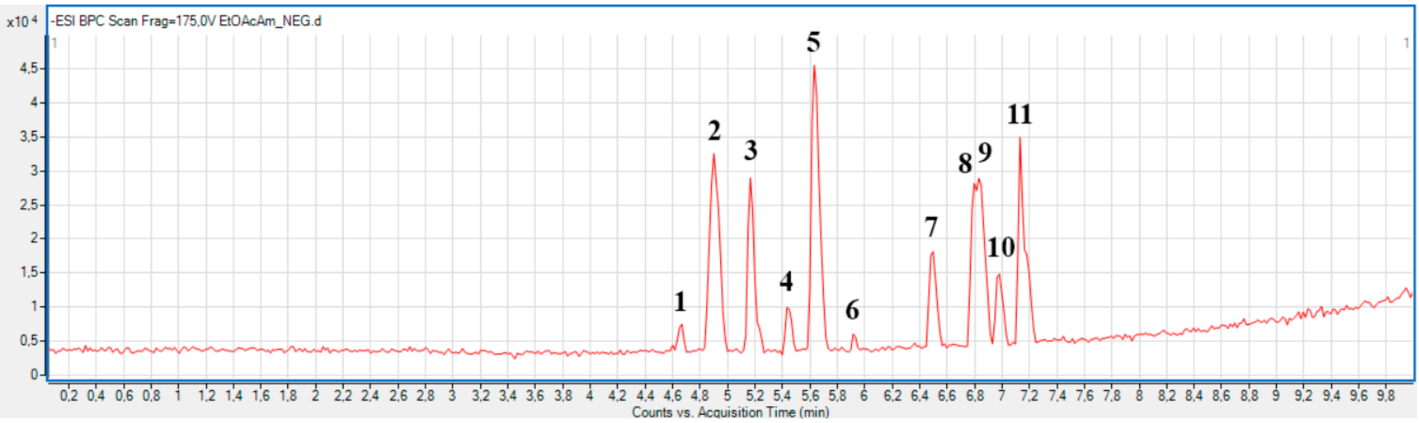

Figure S1. ESI-MS chromatogram of the ethyl acetate fraction (EtOAC.f) from *Annona muricata* leaf (negative mode). Numbers corresponding to major peaks and their respective compounds are shown in Supplementary Table S1.

Peak	Compound (tentative identification)	Retention time (min)	Formula [M-H]$^-$	Mass calculated for [M-H]$^-$	*m/z* of [M-H]$^-$	Error (ppm)	*m/z* of fragments of [M-H]$^-$	References
1	Chlorogenic acid	4.6	$C_{16}H_{17}O_9^-$	353.0878	353.0864	3.9	191, 161, 127, 125	doi: 10.3390/12030679
2	Procyanidin B2	4.8	$C_{30}H_{25}O_{12}^-$	577.1351	577.1340	-1.9	451, 407, 289, 203, 161, 125	Justino 2018
3	Procyanidin C1	5.2	$C_{45}H_{37}O_{18}^-$	865.1985	865.1985	0.0	739, 577, 503, 451, 407, 289, 243, 125	Justino 2018
4	Caffeic acid	5.4	$C_9H_7O_4^-$	179.0350	179.0343	3.9	176, 135	doi: 10.3390/ijms13010260
5	(Epi)catechin	5.6	$C_{15}H_{13}O_6^-$	289.0718	289.0711	-2.4	271, 245, 203, 179, 125	Justino 2018
6	Quercetin-diglucoside	5.9	$C_{27}H_{29}O_{17}^-$	625.1410	625.1409	-0.1	463, 301, 300, 179	Justino 2018
7	Quercetin-glucosyl-pentoside	6.5	$C_{26}H_{27}O_{16}^-$	595.1305	595.1304	-0.1	427, 301, 300, 271, 229, 179	Justino 2018
8	Quercetin-glucoside	6.8	$C_{12}H_{19}O_{12}^-$	463.0877	463.0875	-0.4	301, 300, 271, 125	Justino 2018
9	Rutin	6.8	$C_{27}H_{29}O_{16}^-$	609.1461	609.1462	0.1	565, 463, 301, 300, 179	Justino 2018
10	Quercetin-pentoside	6.9	$C_{20}H_{17}O_{11}^-$	433.0776	433.0759	-3.9	301, 300, 271, 233, 165	Chen 2012.
11	Quercetin-rhamnoside	7.2	$C_{21}H_{19}O_{11}^-$	447.0933	447.0911	-4.9	341, 301, 300, 285, 284	Justino 2018

Table S1. Compounds identified in the ethyl acetate fraction from *Annona muricata* leaf (EtOAc) by HPLC-ESI-MS/MS (negative mode).

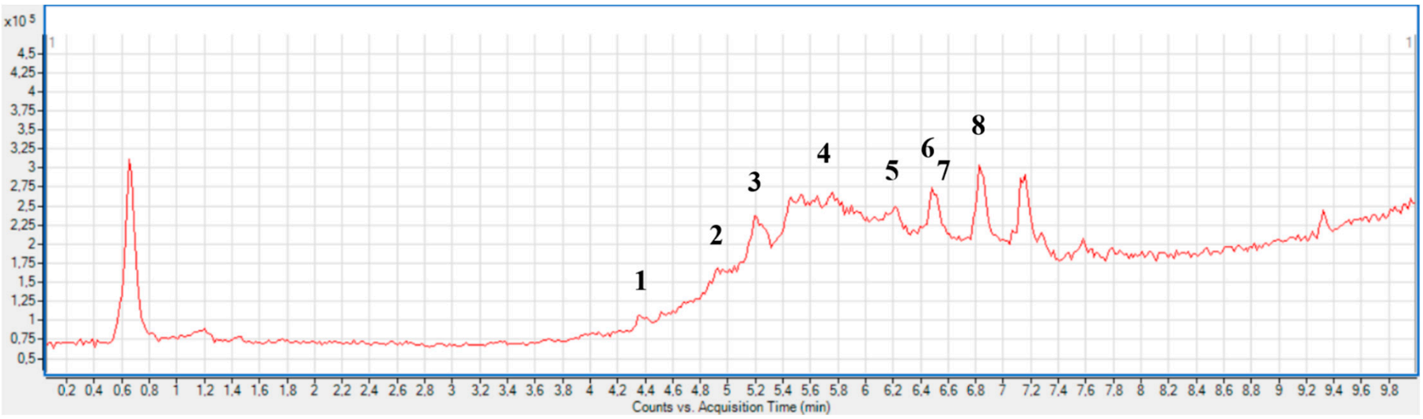

Figure S2. ESI-MS chromatogram of the n-butanol (BuOH.f) from *Annona muricata* leaf (negative mode). Numbers corresponding to major peaks and their respective compounds are shown in Table S2.

Peak	Compound (tentative identification)	Retention time (min)	Formula [M-H]$^-$	Mass calculated for [M-H]$^-$	*m/z* of [M-H]$^-$	Error (ppm)	*m/z* of fragments of [M-H]$^-$	References
1	Chlorogenic acid	4.4	$C_{16}H_{17}O_9^-$	353.0878	353.0864	3.9	191, 161, 127, 125	doi: 10.3390/12030679
2	Procyanidin B2	4.9	$C_{30}H_{25}O_{12}^-$	577.1351	577.1340	-1.9	451, 407, 289, 203, 161, 125	Justino 2018
3	Procyanidin C1	5.2	$C_{45}H_{37}O_{18}^-$	865.1985	865.1985	0.0	739, 577, 503, 451, 407, 289, 243, 125	Justino 2018
4	(Epi)catechin	5.6	$C_{15}H_{13}O_6^-$	289.0718	289.0711	-2.4	271, 245, 203, 179, 125	Justino 2018
5	Quercetin-diglucoside	6.2	$C_{27}H_{29}O_{17}^-$	625.1410	625.1409	-0.1	463, 301, 300, 179	Justino 2018
6	Quercetin-glucosyl-pentoside	6.4	$C_{26}H_{27}O_{16}^-$	595.1305	595.1304	-0.1	427, 301, 300, 271, 229, 179	Justino 2018
7	Quercetin-xyloside-rutinoside	6.4	$C_{32}H_{37}O_{20}^-$	741.1883	741.1851	4,31	653,487, 301, 300	doi: 10.3390/molecules200611490
8	Rutin	6.8	$C_{27}H_{29}O_{16}^-$	609.1461	609.1462	0.1	565, 463, 301, 300, 179	Justino 2018

Table S2. Compounds identified in the n-butanol fraction (BuOH.f) from *Annona muricata* leaf (BuOH.f) by HPLC-ESI-MS/MS (negative mode).
